# High Intensity Aerobic Exercise Training Improves Deficits of Cardiovascular Autonomic Function in a Rat Model of Type 1 Diabetes Mellitus with Moderate Hyperglycemia

**DOI:** 10.1155/2016/8164518

**Published:** 2016-01-18

**Authors:** Kenneth N. Grisé, T. Dylan Olver, Matthew W. McDonald, Adwitia Dey, Mao Jiang, James C. Lacefield, J. Kevin Shoemaker, Earl G. Noble, C. W. James Melling

**Affiliations:** ^1^Exercise Biochemistry Laboratory, School of Kinesiology, Faculty of Health Sciences, Western University, London, ON, Canada N6A 3K7; ^2^Neurovascular Research Laboratory, School of Kinesiology, Faculty of Health Sciences, Western University, London, ON, Canada N6A 3K7; ^3^Department of Electrical and Computer Engineering, Department of Medical Biophysics and Robarts Research Institute, Western University, London, ON, Canada N6A 3K7; ^4^Department of Physiology and Pharmacology, Western University, London, ON, Canada N6A 3K7; ^5^Lawson Health Research Institute, London, ON, Canada N6C 2R5

## Abstract

Indices of cardiovascular autonomic neuropathy (CAN) in experimental models of Type 1 diabetes mellitus (T1DM) are often contrary to clinical data. Here, we investigated whether a relatable insulin-treated model of T1DM would induce deficits in cardiovascular (CV) autonomic function more reflective of clinical results and if exercise training could prevent those deficits. Sixty-four rats were divided into four groups: sedentary control (C), sedentary T1DM (D), control exercise (CX), or T1DM exercise (DX). Diabetes was induced via multiple low-dose injections of streptozotocin and blood glucose was maintained at moderate hyperglycemia (9–17 mM) through insulin supplementation. Exercise training consisted of daily treadmill running for 10 weeks. Compared to C, D had blunted baroreflex sensitivity, increased vascular sympathetic tone, increased serum neuropeptide Y (NPY), and decreased intrinsic heart rate. In contrast, DX differed from D in all measures of CAN (except NPY), including heart rate variability. These findings demonstrate that this T1DM model elicits deficits and exercise-mediated improvements to CV autonomic function which are reflective of clinical T1DM.

## 1. Introduction

A common and serious complication of Type 1 diabetes mellitus (T1DM) is diabetic autonomic neuropathy [1, 2]. Cardiovascular autonomic neuropathy (CAN) is a subset of diabetic autonomic neuropathy characterized by impaired autonomic control of the cardiovascular (CV) system [3]. CAN is also consistently associated with increased mortality. For instance, CAN has been reported to increase the mortality of diabetic patients by a factor of 3.45 [4]. Clinically, the most common methods for assessing CAN are heart rate variability (HRV) analysis and baroreflex sensitivity (BRS) [3, 5, 6]. In T1DM, aspects of the baroreflex arc can be impaired [7], such that both baroreceptor activity and excitability are blunted [8, 9] and the aortic depressor nerves undergo axonal atrophy [8]. As well, autonomic efferents, primarily of the parasympathetic nervous system (PSNS), have decreased activity, reduced responsiveness, and decreased neurochemical activity in the heart [10, 11]. Impairment of central nervous system regions has also been reported as the limiting factor of BRS [12, 13]. Reduced heart rate variability (HRV) is often the earliest symptom of CAN [14]. Whether measured by time domain analysis or by frequency domain analysis and whether in clinical or experimental T1DM, HRV is consistently reported to be reduced in T1DM [5, 14–17].

Exercise has been demonstrated to be an effective means of improving deficits in HRV and BRS in both clinical and experimental T1DM [18–21]. Such improvements have been attributed to improved insulin sensitivity, increased endogenous antioxidant and anti-inflammatory mediators, and improved autonomic control of the CV system [22–24]. Despite similar reductions in HRV and BRS, there are marked differences in early-stage changes to other CV parameters between clinical and experimental T1DM [25]. Specifically, in clinical T1DM, increases in heart rate (HR) and blood pressure (BP) are commonly reported in early autonomic neuropathy [1, 3, 14, 24, 26–28]. In contrast, experimental STZ-induced T1DM is regularly associated with decreased BP and HR, beginning shortly after diabetes induction [15, 16, 29–31]. Due to these opposing initial changes in BP and HR, exercise training is often observed to produce contrasting outcomes on CV parameters in experimental and clinical T1DM, namely, increased BP and HR in experimental T1DM and decreased BP and HR in clinical T1DM [19, 25, 32–34]. As a result, both the increase and decrease of these CV factors are concurrently cited as exercise-mediated improvements to CAN with little consideration of the fact that the changes are opposed between these two contexts of T1DM [25]. This is important because if animal models do not accurately reproduce T1DM pathology, then the outcomes of experimental studies may not translate to the treatment of human CAN, as the mechanisms underlying the pathology and exercise modifications may differ.

Another important difference between experimental and clinical T1DM is the common omission of insulin treatment in experimental diabetes leading to severe hyperglycemia ranging from roughly 17 to 25 mM blood glucose concentrations ([BG]) [15, 16, 29, 30]. As the severity and duration of hyperglycemia have been shown to influence the degree of diabetic neuropathy, acute and steep elevations of [BG] in STZ-induced T1DM may not only cause early onset neuropathy to the PSNS but also cause acute neuropathy of the sympathetic nervous system (SNS) and directly affect the sinoatrial (SA) node. These changes may mediate the observed reduction in BP and HR that arise acutely in experimental T1DM and in late-stage clinical T1DM [2, 35–38]. Indeed, intensive insulin therapy has been shown to restore BP and HR to non-T1DM levels in STZ-induced T1DM rats [25, 39].

Yet, despite the use of insulin therapy in clinical T1DM, it is often the case that chronic, moderate hyperglycemia is maintained as a result of difficulties in regulating [BG] in response to dynamic influences on glycemic control, such as food intake and exercise [40, 41]. This is often resultant of a tendency to err on the side of moderate hyperglycemia in order to circumvent the acute discomfort and danger associated with hypoglycemic episodes, which occur more frequently with diabetic neuropathy due to the impairment of the glucagon response [40, 42, 43]. To address this, our laboratory established a model of T1DM using a multiple low-dose STZ-treatment and insulin therapy to replicate the moderate hyperglycemia observed in clinical T1DM [44]. In our previous studies that employed this model, we observed impairments in glucose tolerance, vascular responsiveness, cardiac function, and bone health, which were improved with high intensity aerobic exercise training [44–47].

The purpose of the current study was to investigate whether our model of multiple low-dose STZ-induced T1DM with insulin therapy would induce deficits in cardiovascular autonomic function more representative of clinical T1DM, and if high intensity aerobic training could prevent those deficits. We hypothesised that (1) our model of STZ-induced T1DM would elicit indices of CAN, including a blunted BRS (bradycardia and tachycardia response), lowered HRV and intrinsic heart rate, increased vascular sympathetic tone, and increased mean arterial pressure, and (2) high intensity aerobic exercise training would prevent or ameliorate the indications of CAN.

## 2. Materials and Methods

### 2.1. Ethics Approval

The protocols used in this investigation were approved by the University of Western Ontario Council on Animal Care and conformed to the guidelines of the Canadian Council on Animal Care.

### 2.2. Animals

Eight-week-old male Sprague-Dawley rats were obtained from Charles River Laboratories Canada (Saint-Constant, Quebec). The rats were housed in pairs and maintained on a 12-hour dark/light cycle at a constant temperature (20 ± 1°C) and relative humidity (50%). Rats were allowed access to standard rat chow and water* ad libitum*.

### 2.3. Experimental Groups

Sixty-four rats were randomly assigned to one of four groups as follows: (1) sedentary control (C, *n* = 16); (2) exercised control (CX, *n* = 16); (3) sedentary T1DM (D, *n* = 16); (4) exercised T1DM (DX, *n* = 16). All functional and blood endpoint measures were acquired 24 hours after the final exercise bout.

### 2.4. T1DM Induction and Insulin Dose

Upon arrival rats were acclimatized to the laboratory setting for five days. Subsequently, T1DM was induced over five consecutive days by multiple intraperitoneal (IP) injections of 20 mg/kg streptozotocin (STZ, Sigma-Aldrich) dissolved in a citrate buffer (0.1 M, pH 4.5). Diabetes was confirmed by blood glucose measurements greater than or equal to 18 mM on two consecutive days. If necessary, subsequent 20 mg/kg STZ injections were administered until diabetes was confirmed. Following the confirmation of diabetes, insulin pellets (1 pellet; 2 U insulin/day; Linplant, Linshin Canada, Inc., Toronto, Ontario, Canada) were implanted subcutaneously in the abdominal region. Insulin pellet doses were then monitored for 1 week and adjusted (±0.5 pellets) in order to obtain daily nonfasting blood glucose concentrations in the moderate hyperglycemic range of 9–17 mM. Insulin dose was determined by multiplying the total quantity of pellet implanted (0.5 pellet increments) by the amount of insulin released per pellet (2 units of insulin/day/pellet) divided by the body weight (Kg) of the rat.

### 2.5. Body Weight and Blood Glucose Concentration

Body weights and nonfasting blood glucose concentrations were obtained weekly. Blood was obtained from the saphenous vein by venous puncture with a 30-gauge needle and measured via Freestyle Lite Blood Glucose Monitoring System (Abbott Diabetes Care Inc., Mississauga, Ontario, Canada).

### 2.6. Intravenous Glucose Tolerance Test

Intravenous glucose tolerance tests (IVGTT) were performed on all animals prior to T1DM induction (pre-T1DM) and at the end of week 10 of the exercise training period. Rats were fasted for approximately 8 to 12 hours prior to the assay and did not perform exercise on the day of their IVGTT. A sterile-filtered dextrose solution (50% dextrose, 50% ddH_2_O) was injected (1 g/kg) into the lateral tail vein of the conscious rat. Following dextrose infusion, blood glucose was measured at 5 minutes, at 10 minutes, and then at 10-minute intervals thereafter until blood glucose levels plateaued.

### 2.7. Exercise Protocol

Prior to the initiation of the exercise training program, rats were familiarized with the exercise equipment on two consecutive days. The familiarization consisted of two 15-minute sessions of running at progressive treadmill speeds up to 30 meters per minute (m/min). The treadmill was a custom-built apparatus fabricated by the physical plant at University of Western Ontario and has been used in many previous studies [44–47]. The exercise training program consisted of 1 hour of motor-driven treadmill running per day at 27 m/min with a 6-degree incline, 5 days per week, for 10 weeks. The exercise intensity was determined based on earlier research that investigated oxygen uptake in rats at various treadmill speeds. The chosen intensity was found to represent approximately 75–85%  VO_2max_ [48, 49].

### 2.8. Preparative Surgery and Instrumentation

To achieve a surgical plane of anesthesia, rats were placed in an induction chamber circulating 4% isoflurane (96% O_2_). Once motor reflexes were undetectable, rats were transferred to a nosecone delivering 3% isoflurane (97% O_2_) and placed on a hot water pad (37°C). Rats were cannulated with saline-infused polyethylene (PE90) catheters in the right jugular vein and carotid artery and each catheter was attached to a three-way stopcock. The jugular vein catheter was used for drug infusions and the carotid artery catheter was connected in series with a pressure transducer (PX272, Edwards Life Sciences, Irvine, California, USA) for arterial blood pressure measurements.

At the end of the preparative surgery, rats were injected IP with a 25 mg/Kg “cocktail” of urethane (16 mg/mL) and *α*-chloralose (100 mg/mL), an anesthetic cocktail that has been shown to have the least inhibition of baseline CV control and autonomic function [50]. A total of 10 mL of urethane/*α*-chloralose was made, 5 mL of which was diluted to 50% with ddH_2_O and was used as needed to maintain anesthesia throughout data collection. Isoflurane anesthesia was gradually removed, whereby urethane/*α*-chloralose was the primary anesthesia used during data collection.

### 2.9. Basal Heart Rate, Systolic Blood Pressure, and Mean Arterial Pressure

Heart rate (HR), systolic blood pressure (SBP), and mean arterial pressure (MAP) were determined from the blood pressure pulse waveform and were collected while the rats were under urethane/*α*-chloralose anesthesia in the supine position. The pressure transducer was calibrated using a standard analog manometer. Data were obtained using a PowerLab data acquisition system, digitized, and recorded at 1000 Hz using the bundled LabChart 7 Pro software (ADInstruments, Colorado Springs, CO, USA).

### 2.10. Heart Rate Variability

Prior to drug infusions, 5 minutes of spontaneous electrocardiogram data was sampled at 1000 Hz and analyzed with LabChart HRV analysis software (ADInstruments). Time domain analysis of the standard deviation between normal peak pulses of the pressure pulse waveform (SDNN) was quantified as a measure of the total variability of the HR. Frequency domain analysis of the high frequency (HF) band of the Fast Fourier Transform (FFT) of the data was assessed as an index of parasympathetically mediated HRV.

### 2.11. Baroreflex Sensitivity

Baroreflex sensitivity (BRS) was assessed using the modified Oxford technique [51, 52]. The BRS was quantified using the slope of the linear regression line representing the linear portion of the sigmoidal heart rate-systolic blood pressure relationship (ΔHR {BPM}/ΔSBP {mmHg}^−1^) after rapid bolus injections (~5 s) of phenylephrine (PE, 12 *μ*g/Kg, 10 *μ*g/mL) and sodium nitroprusside (SNP, 60 *μ*g/Kg, 110 *μ*g/mL) dissolved in ddH_2_O. The rationale for this method is detailed by Studinger et al. (2007) [54]. For each drug, the catheter was first filled with a 0.2 mL volume to ensure accuracy of the drug dose. After a stable baseline was obtained, a bolus injection of SNP was rapidly infused and the reflex SNS mediated tachycardia response was measured. The analysis began at the onset of SBP decrease after SNP infusion and ended when SBP reached its nadir. This was followed by a saline flush to washout any remaining SNP in the catheter. After a stable baseline was reestablished, this same procedure was then followed using PE to measure PSNS mediated reflex bradycardia, except that analysis began at the onset of SBP increase and ended when SBP reached its zenith. Responses to PE and SNP were plotted separately and only regression lines (slopes) with correlation coefficients (*r*) ≥ 0.70 and *p* < 0.05 were accepted [53, 54].

### 2.12. Vascular Sympathetic Tone

To measure the sympathetic contribution to baseline vascular resistance, MAP was assessed before and after a bolus injection of the *α*-adrenergic receptor blocker, prazosin (85 *μ*g/Kg, 500 *μ*g/mL). Following this protocol, animals were euthanized via exsanguination while still under urethane/*α*-chloralose anesthesia.

### 2.13. Neuropeptide Y ELISA

To ensure physiological testing did not confound serum neuropeptide Y concentration [NPY] measurement, a subset of animals from each group did not undergo surgery for heart rate variability, baroreflex sensitivity, mean arterial pressure, or vascular sympathetic tone measurements. Rather, at the end of the 10-week exercise training period these animals were anesthetized via intraperitoneal injection of sodium pentobarbital (65 mg/kg) and blood serum samples for [NPY] measurement were collected upon euthanasia. Serum [NPY] was measured using an NPY ELISA kit (USCN Life Sciences Inc.) according to the manufacturer's instructions.

### 2.14. Intrinsic Heart Rate

A Langendorff preparation was used to measure intrinsic heart rate. Following the euthanasia of animals for blood [NPY] measurement, hearts were extracted and immediately arrested by placing them in ice cold Krebs-Henseleit buffer (KHB). Hearts were cannulated for unpaced retrograde aortic constant flow perfusion (15 mL/min) of coronary arteries with KHB (containing 120 mM NaCl, 4.63 M KCl, 1.17 mM KH_2_PO_4_, 1.25 mM CaCl_2_, 1.2 mM MgCl_2_, 20 mM NaHCO_3_, and 8 mM glucose gassed with 95% O_2_ and 5% CO_2_) that was maintained at 37°C [55]. Hearts were equilibrated for 30 min to determine baseline intrinsic heart rate.

### 2.15. Data Analysis and Statistics

Body weight and blood glucose concentrations were compared using a two-way repeated measures ANOVA, while endpoint measures were compared by two-way ANOVA, with the exception of endpoint insulin dose, which was compared using a one-tailed *t*-test. When significance was found, pairwise comparisons were made using the Fisher LSD post hoc test. Data are represented as mean ± standard error, with a significance level set at *p* < 0.05.

## 3. Results

### 3.1. Animal Characteristics

**Figure 1:**
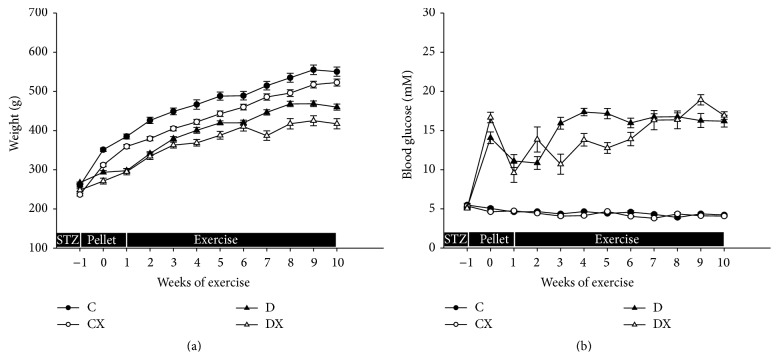
(a) Weekly body weights: C, sedentary control (*n* = 16); CX, control exercise (*n* = 16); D, sedentary T1DM (*n* = 15); DX, T1DM exercise (*n* = 12). (b) Weekly blood glucose concentrations: C (*n* = 16); CX (*n* = 15); D (*n* = 15); DX (*n* = 13). STZ, pellet, and exercise indicate the periods of STZ injection, insulin pellet implantation, and aerobic exercise, respectively. Significantly different groups (*p* < 0.05). Data are mean ± SE. There was significant difference in body weight between T1DM and non-T1DM groups, while a significant difference was evident between exercised and nonexercised groups. The blood glucose concentrations in the T1DM groups were significantly different than non-T1DM groups.

All groups increased in body weight over the course of the study (*p* < 0.05, Figure 1(a)). At the end of the study, the body weights of the T1DM groups (D and DX) were lower than non-T1DM groups (C and CX), and exercised groups (CX and DX) weighed less than their nonexercised counterparts (C and D; *p* < 0.05). Following the confirmation of diabetes, weekly [BG] was mostly maintained in the targeted range of 9–17 mM; however, the [BG] did move outside of this range periodically. The [BG] in the T1DM groups were elevated in comparison to the non-T1DM groups (*p* < 0.05; Figure 1(b)). Within the non-T1DM and T1DM groups, there was no difference in [BG] between nonexercised and exercised groups (C versus CX and D versus DX; *p* > 0.05).

### 3.2. Intravenous Glucose Tolerance Test and Insulin Dosages

**Figure 2:**
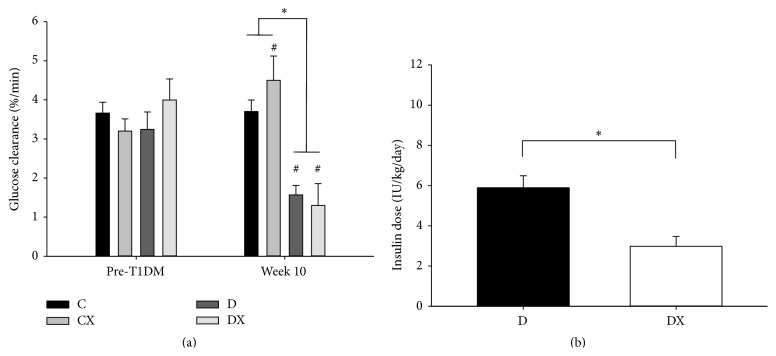
(a) IVGTT glucose clearance rate (*K*_G_) values prior to T1DM induction (pre-T1DM) and week 10 of exercise training: C, sedentary control (*n* = 16); CX, control exercise (*n* = 15); D, sedentary T1DM (*n* = 15); DX, T1DM exercise (*n* = 15). (b) Insulin dosages at week 10: D (*n* = 16); DX (*n* = 16). ^*∗*^Significantly different groups (*p* < 0.05). ^#^Significantly different from week 1. Data are mean ± SE.

The glucose clearance rate (*K*_G_) of the diabetic groups (D and DX) decreased from pre-T1DM to week 10 of training (*p* < 0.05), whereas *K*_G_ of the CX group increased (*p* < 0.05; Figure 2(a)). Both diabetic groups had significantly lower *K*_G_ values than both the control groups (C and CX) at week 10 (*p* < 0.05; Figure 2(a)). However, there was not a significant interaction between diabetes and exercise on *K*_G_. The amount of insulin supplementation that the DX group received was significantly less than the amount the D group received at week 10 (*p* < 0.05; Figure 2(b)).

### 3.3. Mean Arterial Pressure, Heart Rate, and Intrinsic Heart Rate

**Figure 3:**
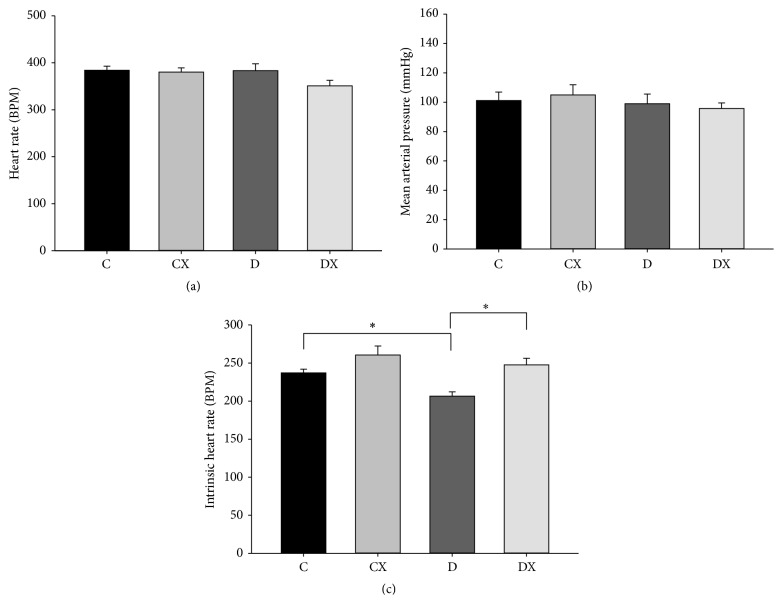
(a) Heart rate (beats per minute) and (b) mean arterial pressure at week 10. C, sedentary control (*n* = 7); CX, control exercise (*n* = 7); D, sedentary T1DM (*n* = 8); DX, T1DM exercise (*n* = 10). (c) Intrinsic heart rate (IHR) at week 10. C (*n* = 10); CX (*n* = 11); D (*n* = 12); DX (*n* = 9). ^*∗*^Significantly different groups (*p* < 0.05). Data are mean ± SE.

For resting HR and MAP, there was not a significant difference between groups at week 10 (Figures 3(a) and 3(b), resp.). However, for the intrinsic heart rate (IHR), there was main effect of both exercise and T1DM, where T1DM decreased the IHR, while exercise increased IHR (*p* < 0.05, Figure 3(c)). Further, within the T1DM groups (D and DX), exercise increased IHR, while within the nonexercised groups (C and D) T1DM decreased IHR (*p* < 0.05).

### 3.4. Heart Rate Variability

**Figure 4:**
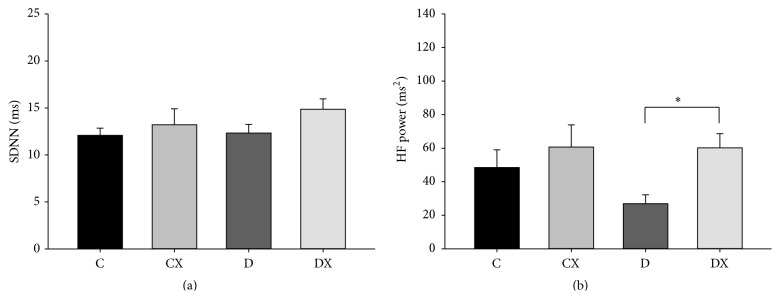
(a) Total HRV (SDNN) at week 10: C, sedentary control (*n* = 5); CX, control exercise (*n* = 7); D, sedentary T1DM (*n* = 8); DX, T1DM exercise (*n* = 8). (b) High Frequency (HF, parasympathetic) HRV component at week 10: C (*n* = 6); CX (*n* = 7); D (*n* = 8); DX (*n* = 8). ^*∗*^Significantly different groups (*p* < 0.05). Data are mean ± SE.

Total HRV at week 10, as measured by the standard deviation of the normal pulse wave peaks (SDNN), was not significantly different between groups (Figure 4(a)). However, there was a main effect of exercise on the HF contribution to HRV, where exercise increased HF HRV (*p* < 0.05, Figure 4(b)). Particularly, within the T1DM groups (D and DX), exercise increased HF HRV (*p* < 0.05).

### 3.5. Baroreflex Sensitivity

**Figure 5:**
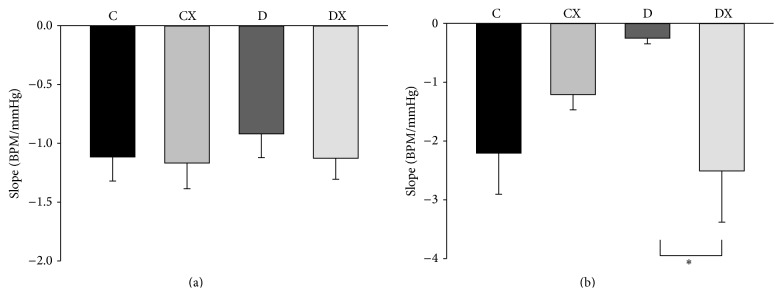
(a) Tachycardia baroreflex response sensitivity to sodium nitroprusside at week 10: C, sedentary control (*n* = 7); CX, control exercise (*n* = 6); D, sedentary T1DM (*n* = 6); DX, T1DM exercise (*n* = 10). (b) Bradycardia baroreflex response to phenylephrine at week 10: C (*n* = 7); CX (*n* = 5); D (*n* = 7); DX (*n* = 10). ^*∗*^Significantly different groups (*p* < 0.05). Data are mean ± SE.

In response to SNP infusion, there was not a significant difference between groups in the tachycardia BRS response (Figure 5(a)). However, a significant interaction between T1DM and exercise was observed for BRS during the bradycardia response to phenylephrine (*p* < 0.05, Figure 5(b)). More specifically, within the T1DM groups (D and DX) exercise prevented the reduction in BRS that was observed in the D group (*p* < 0.05).

### 3.6. Vascular Sympathetic Tone and Serum NPY

**Figure 6:**
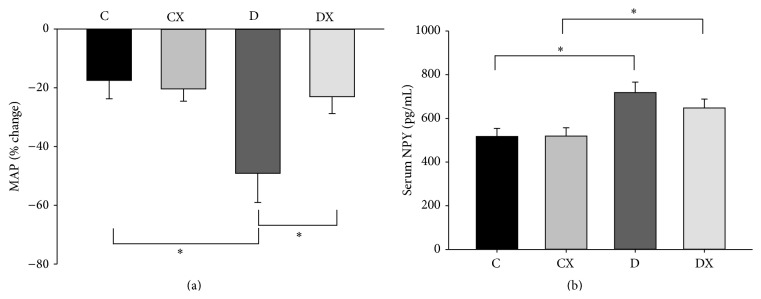
(a) Vascular sympathetic tone (VST) at week 10. This was determined by measuring the percent change in MAP after prazosin treatment at week 10: C, sedentary control (*n* = 8); CX, control exercise (*n* = 7); D, sedentary T1DM (*n* = 5); DX, T1DM exercise (*n* = 7). (b) Serum NPY at week 10: C (*n* = 6); CX (*n* = 6); D (*n* = 6); DX (*n* = 6). ^*∗*^Significantly different groups (*p* < 0.05). Data are mean ± SE.

An interaction between T1DM and exercise was observed for the prazosin-induced change in MAP (*p* < 0.05, Figure 6(a)). Within the nonexercised groups (C and D), T1DM resulted in an increased change in MAP (*p* < 0.05). Within the T1DM groups (D and DX) exercise prevented the increased change in MAP observed in the D group (*p* < 0.05). There was also a main effect of T1DM on [NPY] (*p* < 0.05, Figure 6(b)). Within the nonexercised groups (C and D) and exercised groups (CX and DX), serum [NPY] was increased by T1DM (*p* < 0.05).

## 4. Discussion

This study demonstrated that a multiple low-dose STZ model with moderate hyperglycemia, maintained using insulin therapy, produced deficits in cardiovascular autonomic function without inducing the resting bradycardia or hypotension typical of other STZ models. This study also showed that high intensity aerobic exercise training can prevent deficits of cardiovascular autonomic function caused by T1DM. Furthermore, because [BG] was held within a moderate hyperglycemic range, the observed exercise-mediated improvements to indications of CAN were independent of changes in [BG] and, instead, may primarily have been the result of improvements to other aspects of glucoregulation and/or the preservation of autonomic nervous system function.

Although we found time domain analysis of total HRV, as measured by the SDNN, did not demonstrate differences between groups, frequency domain analysis exposed a reduction in the HF power in the D group compared with the DX group. Since the HF power corresponds to the level of vagally mediated parasympathetic HRV, these results demonstrate not only the detrimental effects of T1DM on autonomic cardiac control but also the benefits of exercise training toward ameliorating those effects. These findings are similar to those of other experiments of both experimental [16, 21] and clinical diabetes [18, 56, 57]. For example, Mostarda et al. (2009) reported that STZ-induced T1DM reduced the HF component of HRV, which was improved by exercise [21]. Also, they found that the vagal tonus of the control exercised rats did not differ from sedentary controls [21]. Likewise, Chen et al. (2008) reported that children with T1DM who performed a high level of physical activity did not differ from controls in HRV; however, children with T1DM who had low level of physical activity had significantly reduced HRV compared to both active children with T1DM and non-T1DM children [18]. Thus, the current study provides support that exercise can be an effective means to improve HRV in T1DM.

Both tachycardia and bradycardia responses were studied in the context of BRS analysis in order to explore the control features related to unloading or loading of the baroreceptors, respectively. Some discrepancy exists between different experimental models of T1DM and their impact on BRS measures. Investigations using the hyperglycemic Non-Obese Diabetic (NOD) T1DM mouse model have shown elevations in BRS measures rather than attenuated responses [58]. In contrast, tachycardic-SNP and bradycardic-PE responses have been shown to be lower in STZ-induced T1DM hyperglycemic rats in comparison to non-T1DM controls [21] but were improved with exercise training [59]. In the current study, the slope of the hypotensive tachycardia response was not significantly different between any of the groups suggesting that responses to baroreceptor unloading are not affected by T1DM or exercise. However, T1DM reduced the bradycardia response to baroreceptor loading, which was nullified by concurrent exercise training. These findings are in line with previous reports demonstrating a bradycardia change in PE-BRS without an accompanying change in SNP-BRS [60], which was improved following aerobic exercise [61]. Discrepancies in BRS responses in T1DM models seem to be closely associated with both the duration and the severity of diabetes. A recent study examining the time-course of BRS changes in response to STZ-induced hyperglycemia reported that alteration of the SNP-BRS was not evident until 12 weeks of diabetes, while a change in PE-BRS was evident as early as 4 weeks after induction [62]. Interestingly, the animals in the aforementioned study were moderately hyperglycemic (16–18 mM), suggesting that the severity of the hyperglycemia may play a role in the progression of this neuropathy. This relationship has also been demonstrated in humans. Vinik and Ziegler (2007) reported that poor glycemic control [63] and duration of diabetes [64] play a central role in progression of cardiovascular autonomic neuropathy. Yet, it is not clear what role insulin therapy may play in the neuropathy. Insulin supplementation to STZ-induced T1DM rats can modify the changes in BRS sensitivity evident at 48 weeks of T1DM [65]. Indeed, in clinical T1DM patients, intensive therapy is well documented to slow the progression and delay the appearance of abnormal autonomic function [66].

However, the current study provides evidence that the ability of exercise to ameliorate cardiovascular autonomic dysfunction may be independent of its ability to reduce [BG], which challenges the direct relationship between [BG] and CAN suggested by previous studies [67–69]. The IVGTT performed at the conclusion of the 10-week exercise period demonstrated an increased glucose clearance rate (*K*_G_) and therefore glucose tolerance, in the CX group compared to the preexercise training period. However, in both the sedentary and exercise diabetic groups there was an equal decline in *K*_G_ to nearly the same rate. This decrease was significantly different from pre-T1DM values and the week 10 values of the C and CX groups. While this would normally indicate that both of the diabetic groups developed equally impaired glucose tolerance, it was also the case that the DX group required approximately half of the dosage of exogenous insulin compared to the D group to maintain their [BG] in the 9–17 mM range. With double the insulin dose, it is likely that the total serum insulin over a given time during IVGTT would have been greater in the D than DX group, and with their *K*_G_ being equal, that would indicate that there was a greater insulin sensitivity in the diabetic exercise group [70, 71]. Together, these IVGTT results demonstrate that exercise training improved glucose tolerance and insulin sensitivity [67]. Furthermore, since the [BG] of the diabetic groups in this study was held in a constant range, any abovementioned exercise-induced improvements to CV autonomic function would not have been mediated through a reduction in systemic [BG] but may have been the result of improvements in insulin sensitivity and glucose utilization [72, 73]. This should be borne in mind when considering the effects of diabetes and exercise on indices of CV autonomic function, such as HRV and BRS.

An alternative mechanism by which exercise can influence BRS was reported by Bernardi et al. (2011), who elucidated the importance of tissue oxygenation in T1DM [74]. They demonstrated that a reduced parasympathetic BRS in patients with T1DM was improved by both oxygen supplementation and deep breathing to the same degree, which indicated the increased respiration and oxygen delivery resultant of exercise could have been mediating increases in BRS. This led the authors to suggest that hypoxia in T1DM functionally restrains parasympathetic activity. However, reduced BRS could also be attributed to defects in the baroreceptors, baroreceptor afferent nerves, CNS structures, or efferent fibres of the baroreflex circuit [7, 8, 61]. In the present study, the finding that the tachycardia response of the baroreflex was unimpaired by T1DM, while the bradycardia response was, suggests that the afferent arm and central regulators of the baroreflex were not dysfunctional and that the observed decrement of baroreflex bradycardia may have been caused partly by alterations in efferent parasympathetic outflow [8, 29]. The smaller HF HRV in the D group is consistent with this interpretation.

Another interesting outcome of the current study was the alteration of sympathetic vasomotor control in the D group, which was also modified by concurrent exercise training. In this study, prazosin treatment resulted in a drop in MAP that was approximately twofold greater in the D group compared to the C and DX groups, which is indicative of a much greater sympathetic contribution to the maintenance of baseline vascular resistance [75, 76]. Similarly, Martinez-Nieves and Dunbar (1999) reported that male T1DM rats had a greater decrease in MAP after a bolus injection of prazosin compared to their control cohorts [77]. However, they postulated that an elevated prazosin response could be the result of increased *α*_1_-adrenergic receptor sensitivity [77]. Yet, in this study, the finding that treatment with PE, an *α*_1_-adrenergic receptor agonist, did not result in a greater peak SBP, nor a greater percent increase in SBP from baseline in the T1DM group (data not shown), argues against a receptor-based sensitivity mechanism and, rather, suggests that efferent sympathetic outflow may have been elevated in the D group. However, we cannot determine the mechanism that resulted in prazosin showing a preferential decrease in MAP in the D group versus DX or C based on the data in this study. Yet, in line with the current results, such elevations in resting sympathetic activity would make activation of the BRS response to SNP-induced hypotension more difficult.

The conclusion above regarding sympathetic hyperactivity in the D group is supported by measurements of neuropeptide Y [NPY] obtained in this study. [NPY] is coreleased with norepinephrine from perivascular and cardiac sympathetic nerve terminals during sympathetic activation [78, 79]. In clinical T1DM, a diabetes-related decrease in [NPY] is attributed to impaired sympathetic function, whereas increased [NPY] is attributed to sympathetic overactivity [79–81]. In the current study, serum [NPY] was greater in both of the T1DM groups in comparison to their control groups. This finding is consistent with elevated sympathetic outflow in clinical T1DM [81]. Interestingly, no major impact of exercise was observed on serum [NPY]. Thus, despite the ability of exercise to preserve reflex cardiac function in T1DM, hyperglycemia itself appears to have impacted basal vascular adrenergic activity in both T1DM groups. This observation is consistent with the sympathoexcitatory effect of hyperglycemia [82]. As both T1DM groups were maintained at equally elevated [BG], there may have been a correspondingly similar stimulation of peripheral sympathetic activation and NPY release [79, 82, 83].

Despite improvements by exercise training to deficits of cardiovascular autonomic function, no observable statistical differences in either MAP or HR were evident between any of the groups. Indeed, it has been shown that alterations in autonomic function occur before or without alterations in MAP and HR and are uncorrelated to changes in sympathetic tone [84]. The observed changes in basal sympathetic activity may assist in the maintenance of blood pressure, ventricular function, and cardiac output during the early stage of diabetes, which is supported by our findings that inhibition of sympathetic activity results in a greater decrease in MAP in diabetic rats than normal rats [85]. In that respect, we previously reported that although T1DM animals demonstrated significant alterations in myocardial dimensions and structure, measurements of cardiac performance (ejection fraction, fractional shortening, and cardiac output measurements) were unchanged [44].

To evaluate the heart rate of these animals without neural influence, we measured the beat rate of denervated hearts using the isolated Langendorff technique. We found that the IHR of the D group was lower than both C and DX groups, which would support the notion that decreased IHR masked the effects of sympathetic overactivity in the current study. Further, it supports evidence that STZ-induced diabetes may have a direct effect on heart rate by modifying the heart itself [86, 87]. Interestingly, in some studies, insulin therapy was only able to partially reverse bradycardia and it was shown that STZ-treatment itself could lengthen the action potential duration in the SA node, slowing the HR [88]. However, if hyperglycemia or STZ directly affected cardiac muscle or the SA node and caused a decreased IHR in the D group, it is also the case that exercise training rescued or prevented the deficit, as the IHR of the DX group was not different from the CX group. Thus, previous experimental T1DM studies that reported that STZ-induced bradycardia and hypotension were caused by CAN, and that exercise-induced normalization of HR and BP was evidence of improvements in autonomic function, may really have been observing changes in intrinsic cardiac function which were independent of autonomic control. Such changes could instead have been due to depressed sarcoplasmic reticulum function or impaired calcium handling [87, 89, 90]. Therefore, the direct effects of STZ on the heart and IHR require further examination and should be taken into consideration in future studies that investigate the autonomic regulation of CV function in STZ-induced T1DM models.

An important consideration regarding the design of the current study was the use of anesthetized rats. In order to accurately reflect cardiovascular parameters in such a state, we selected an anaesthetic regime that provides the lowest level of influence on baseline and reflexive CV control attainable in rodent models [50]. A light plane anesthesia 0.5–1.2 g/kg has been shown to maintain the integrity of the cardiovascular system, where higher doses of urethane (above 1.5 g/kg) can produce hypotension and bradycardia, as well as high rates of mortality [91, 92]. In the current study we used a minimal dose of 25 mg/kg, which was reported in previous studies by our laboratory to have little influence on neurovascular blood flow measures [93–95]. That being said, it cannot be determined to what extent, if at all, the autonomic nervous system was augmented by the urethane-chloralose treatment in comparison to conscious animals. Further work examining a comparison of our anesthesia regime with freely moving conscious animals (using telemetry devices) will better address this matter.

## 5. Conclusions

In this study, T1DM induced indications of parasympathetic withdrawal, sympathetic overactivity, and, despite a decreased IHR, no change in resting MAP or HR. However, concurrent exercise training with T1DM maintained the sensitivity of the parasympathetically mediated baroreflex bradycardia, prevented an increase in vascular sympathetic tone, maintained a higher bodyweight, and prevented a decrease in IHR. The ability of exercise training to preserve parasympathetic function in this model of T1DM indicates that the exercise-mediated improvements to parasympathetic function are independent of alterations in [BG]. However, the finding that [NPY] remained elevated suggests that hyperglycemia has a direct impact on adrenergic activity. Taken together, our T1DM model of progressive STZ induction and insulin treatment induced autonomic impairments similar to those observed in clinical T1DM and demonstrates the novelty of this model for investigating the effectiveness of high intensity aerobic exercise training as a means to prevent the progression of CAN in T1DM. Thus, although not examined in this study, the mechanisms that underlie the physiological changes caused by T1DM and exercise can be the focus of future investigations using this model.

## Abbreviations

BG: Blood glucose
BP: Blood pressure
BRS: Baroreflex sensitivity
C: Sedentary control
CAN: Cardiovascular autonomic neuropathy
CX: Exercised control
CV: Cardiovascular
D: Sedentary T1DM
DX: Exercised T1DM
HF: High frequency
HR: Heart rate
HRV: Heart rate variability
IHR: Intrinsic heart rate
MAP: Mean arterial pressure
NPY: Neuropeptide Y
PE: Phenylephrine
SBP: Systolic blood pressure
SNP: Sodium nitroprusside
SNS: Sympathetic nervous system
STZ: Streptozotocin
T1DM: Type 1 diabetes mellitus.
